# Recognizing and managing deventilation syndrome in patients with COPD

**DOI:** 10.36416/1806-3756/e20250256

**Published:** 2026-03-05

**Authors:** Inês Carvalho, Maria Jacob, Anabela Marinho

**Affiliations:** 1. Departamento de Pneumologia, Unidade Local de Saúde de São João, Porto, Portugal.; 2. Faculdade de Medicina, Universidade do Porto, Porto, Portugal.

## TO THE EDITOR:

Nocturnal noninvasive ventilation (NIV) has become a cornerstone therapy for chronic hypercapnic respiratory failure in patients with COPD, offering clear benefits in improving blood gases, reducing mortality, and enhancing quality of life.

A common but underrecognized side effect of NIV, frequently reported by patients yet often overlooked by healthcare professionals, is deventilation syndrome (DVS), characterized by acute dyspnea and discomfort upon NIV removal in the morning. The dyspnea is intense and can be accompanied by rapid, shallow breathing, an increased heart rate, the need to sit upright (in coachman’s position), restlessness, and even panic attacks. Symptoms can last for hours.

The concept of DVS was first introduced by Adler et al.,^1^ who described it as acute dyspnea following NIV withdrawal in patients with chronic respiratory failure. Lüthgen et al.^2^ found DVS in 35% of stable COPD patients who used long-term NIV. The authors observed reductions in inspiratory vital capacity and increased airway resistance, as well as demonstrating that pursed-lip-breathing ventilation (PLBV) significantly reduced morning dyspnea and improved vital capacity. Similarly, Schellenberg et al.^3^ reported DVS in up to 58% of inpatients with severe COPD (GOLD classification III-IV), linking it to dynamic hyperinflation, tachypnea, reduced FEV_1_, and hypercapnia. A recent scoping review further highlighted diverse definitions of DVS, showing a prevalence ranging from 28% to 58%, and implicated mechanisms such as patient-ventilator asynchrony, airway pressure settings, and hyperinflation.^4^ Elshof et al.^5^ advanced this understanding, suggesting that DVS is not due to diaphragmatic muscle inactivity post-NIV but rather to persistent static hyperinflation. In that study, patients with DVS showed lower baseline inspiratory capacity, a further post-NIV decline, and increased diaphragmatic neural drive, as measured by surface electromyography. These findings support the hypothesis that in already hyperinflated individuals, additional NIV-induced dynamic hyperinflation may lead to neuromechanical uncoupling and symptomatic deventilation.

Despite its prevalence, DVS is rarely addressed in clinical practice. Its symptoms-often dismissed as baseline morning breathlessness-may lead to unnecessary therapy escalation and reduced adherence to NIV, as well as decreased patient satisfaction and benefit. This underscores the need for improved recognition and practical solutions.

We report the case of a 68-year-old male with GOLD stage III COPD. He was a former smoker, with a BMI of 27 kg/m^2^, who was on nocturnal bilevel NIV for chronic hypercapnia, at an inspiratory positive airway pressure of 24 cmH_2_O, an expiratory positive airway pressure of 6 cmH_2_O, and a backup rate of 16 breaths/min. Each morning after mask removal, he experienced severe dyspnea (Borg scale 7), tachypnea (~ 22 rpm), with symptoms lasting 60-90 min. A diagnosis of obstructive sleep apnea was excluded, and no alternative causes were identified. Management included the following low-complexity interventions: initiating inhalation therapy immediately upon waking; maintaining NIV during a gradual transition to upright posture (“orthostatism”); and enabling the “ramp down” function of the ventilator to avoid abrupt pressure withdrawal. After reevaluation, his symptoms improved significantly (Borg score decreased to 4) and his inspiratory capacity improved. Notably, NIV adherence remained high, and the patient reported improved morning functioning.

This case underscores the importance of recognizing DVS as a potential reversible complication of long-term NIV therapy in COPD. Mechanistically, DVS appears to result from a combination of dynamic hyperinflation, abrupt loss of end-expiratory support, respiratory muscle overload following sudden removal of airway pressure and increased airway resistance.^2^ ^,^ ^3^ This generates transient dyspnea, discomfort, and reduced ventilatory efficiency, particularly in patients with poor pulmonary reserve. The clinical picture improved through targeted ventilator and behavioral adjustments, aligning with prior studies.^2^ ^,^ ^4^ Although strategies such as PLBV-a ventilator mode designed to mimic pursed-lip breathing and prolong expiration-have shown benefit in small exploratory and retrospective studies, reducing morning dyspnea and improving lung function parameters,^2^ ^,^ ^4^ ^,^ ^6^ our case demonstrates that practical, accessible bedside interventions-simple changes to inhalation timing, posture, and ventilator transitions-can yield meaningful benefits in real-world scenarios. In addition, this case underscores the importance of early recognition of DVS. Failure to identify the syndrome may result in misattribution to disease progression or anxiety, prompting inappropriate escalation of therapy or additional diagnostic testing. Furthermore, validating patient-reported symptoms and incorporating a screening question (e.g., “Do you feel worse or breathless after removing your NIV mask in the morning?”) during routine follow-ups could enhance early detection. For recommendations and future directions, we suggest the following:

1. Clinical screening-Incorporate DVS-specific questions during routine NIV follow-ups.
2. Functional testing-Perform bedside spirometry or inspiratory capacity measurements immediately after NIV withdrawal.
3. Ventilator adjustments-Evaluate ramp-down settings, consider limiting inspiratory positive airway pressure and increasing expiratory time, or switching to PLBV mode, if available.
4. Patient education-Inform patients of the transient nature of DVS and teach simple self-management strategies.
5. Research-High-quality randomized trials are warranted in order to standardize DVS definitions and diagnostic criteria; to compare ventilation modes (e.g., PLBV vs. standard); and to investigate long-term impacts on exercise tolerance and quality of life.

Albeit common, DVS is an as yet under-recognized complication of long-term NIV in COPD. It may significantly impair morning function and reduce NIV adherence. Recognition of the syndrome, together with tailored clinical and ventilator strategies, may alleviate symptoms and enhance compliance. We urge clinicians and device developers to address DVS mechanisms and promote research to optimize nocturnal ventilation strategies.
